# Extreme body mass index and survival in newly diagnosed multiple myeloma patients

**DOI:** 10.1038/s41408-022-00782-7

**Published:** 2023-01-12

**Authors:** Urvi A. Shah, Karissa Whiting, Sean Devlin, Rachel Ershler, Bindu Kanapuru, David J. Lee, Sabrin Tahri, Thomas Gwise, Even H. Rustad, Sham Mailankody, Alexander M. Lesokhin, Dickran Kazandjian, Francesco Maura, Daniel Auclair, Brenda M. Birmann, Saad Z. Usmani, Nicole Gormley, Catherine R. Marinac, Ola Landgren

**Affiliations:** 1 Myeloma Service, Department of Medicine, Memorial Sloan Kettering Cancer Center, 1275 York Avenue, New York, NY 10065 USA; 2 Department of Medicine, Weill Cornell Medical College, 400 East 67th Street, New York, NY 10065 USA; 3 Department of Epidemiology and Biostatistics, Memorial Sloan Kettering Cancer Center, New York, 1275 York Avenue, New York, NY 10065 USA; 4 Division of Hematologic Malignancies II, Center for Drug Evaluation and Research, U.S. Food, and Drug Administration, 5901-B Ammendale Road, Beltsville, MD 20705-1266 USA; 5 Department of Medicine, Massachusetts General Hospital, 55 Fruit Street, Boston, MA 02114 USA; 6 Department of Medical Oncology, Dana-Farber Cancer Institute, Boston, MA 02215 USA; 7 Department of Hematology, Erasmus University Medical Center, 3000CA Rotterdam, The Netherlands; 8 Division of Biometrics IX, Center for Drug Evaluation and Research, U.S. Food and Drug Administration, 5901-B Ammendale Road, Beltsville, MD 20705-1266 USA; 9 Department of Cancer Immunology, Institute for Cancer Research, Oslo University Hospital Radiumhospitalet, 0379 Oslo, Norway; 10 Department of Medicine, Lovisenberg Diaconal Hospital, 0456 Oslo, Norway; 11 Department of Medicine, Sylvester Comprehensive Cancer Center at the University of Miami, 1475 NW 12th Avenue, Miami, FL 33136 USA; 12 Multiple Myeloma Research Foundation, 383 Main Avenue #5, Norwalk, CT 06851 USA; 13 Channing Division of Network Medicine, Department of Medicine, Brigham and Women’s Hospital and Harvard Medical School, 181 Longwood Avenue, Boston, MA 02115 USA; 14 Harvard Medical School, 25 Shattuck Street, Boston, MA 02115 USA

**Keywords:** Risk factors, Myeloma, Cancer metabolism

Dear Editor,

The majority of established prognostic factors for multiple myeloma (MM) are not modifiable and include cytogenetic abnormalities, beta-2 microglobulin, lactate dehydrogenase, age, and Eastern Cooperative Oncology Group (ECOG) performance status [1, 2]. There is a lack of evidence-based clinical interventions available to complement existing treatment modalities to improve prognostic trajectories.

Obesity is a potentially modifiable well-established risk factor [3, 4] associated with an increased incidence of MM [5–7]. However, the evidence is less clear for the association of obesity with clinical outcomes after a new MM diagnosis. Two pooled analyses of large prospective cohorts have demonstrated a relationship between increasing BMI and mortality [8, 9], but it is unclear whether the increased mortality among patients with higher BMI is due to increased cancer incidence, decreased survival after diagnosis, or both [8, 9]. The only study to evaluate BMI at the time of MM diagnosis and long-term clinical outcomes analyzed data from 2968 patients in the Veterans Health Administration System. The authors of that study found that obese patients with MM experienced superior survival, except those with substantial weight loss in the year prior to diagnosis and those who were underweight at diagnosis (BMI < 18.5) [10].

The focus of the present study was to investigate the impact of BMI on progression-free survival (PFS) and overall survival (OS) in patients with newly diagnosed MM. We obtained the data from 1142 patients from the Multiple Myeloma Research Foundation CoMMpass registry (NCT01454297, version IA15). CoMMpass is a prospective observational study that followed patients every six months until death or censoring.

BMI was calculated from height and weight recorded at MM diagnosis and classified into: underweight (<18.5 kg/m^2^), normal (18.5–<25 kg/m^2^), overweight (25–<30 kg/m^2^), moderately obese (30–<35 kg/m^2^) and severely obese (≥35 kg/m^2^). Of the 1,135 patients in the CoMMpass registry, 22 were excluded from the analysis for missing data or extreme values (<36 inches). Demographic and clinical covariates were ascertained from baseline data. The Charlson Comorbidity Index (CCI) was calculated utilizing past medical history and adverse event data. A simplified frailty scale was calculated utilizing age, CCI and Eastern Cooperative Oncology Group (ECOG) performance status, and patients were divided into frail and nonfrail as described previously [11].

PFS was defined as time from diagnosis to first progression or death. Patients who did not experience an event were censored at last follow-up. OS was defined as time from diagnosis to death or last follow-up for those who survived.

A multivariable Cox regression model was used to estimate hazard ratios (HR) for the relationship between BMI and PFS, and OS. The model included age, race, sex, International Staging System (ISS), ECOG performance status, cytogenetic risk, induction treatment combination, and autologous stem cell transplantation (ASCT) as covariates. ASCT (specifically, any ASCT before the first progression event) was treated as a time-dependent covariate to adequately adjust for time at risk before receiving ASCT. CCI and frailty score was not included in the main multivariable model as the CCI score was calculated utilizing past medical history and adverse event data, the accuracy of which is dependent on the health care provider’s documentation. Instead, we performed sensitivity analyses, including CCI (removing age) or frailty score (removing age and ECOG) in multivariate models.

Survival curves and median survival were estimated using the Kaplan–Meier method. All analyses were exploratory with no adjustment for multiplicity, and the alpha error level was set at 5% for presenting 95% confidence intervals (CI). Analyses were performed using R version 3.5.1.

Descriptive characteristics of 1120 patients with available BMI are provided in Table 1. Thirty percent had a normal BMI, 38.2% were overweight, 17·9% were moderately obese, 11.9% were severely obese and 2.0% were underweight. The median age at diagnosis did not vary by BMI category (63–64 years) except for the severely obese that had a median age of 61 years. Male patients were more likely to be overweight or obese (73.5%) compared to female patients (59.5%), and females were more likely to be underweight (3.6%) compared to males (0.88%). White (72.1%) and Black patients (70.1%) were more likely to have an elevated BMI compared to Asians (44.4%), other (50%), and unknown race (53.6%). The ECOG performance status was higher at the extremes of BMI; 68% of underweight patients and 58% of severely obese patients had ECOG ≥ 1 compared to 43–51% in normal weight, overweight, or moderately obese patients. In contrast, individuals were less likely to receive carfilzomib-based therapy if they had an elevated BMI (27% normal vs. 18% overweight, 11% moderately obese, 13% severely obese). There were no significant differences in CCI (*p* = 0.21) and frailty score between BMI groups (*p* = 0.4).

**Table 1:** Select baseline demographics according to category of BMI at diagnosis among 1120 newly diagnosed MM patients in the multiple myeloma research foundation CoMMpass registry.

Characteristic	Overall, *N* = 1120^a^	Underweight, *N* = 22^a^	Normal, *N* = 336^a^	Overweight, *N* = 428^a^	Moderately obese (≥30 & <35), *N* = 201^a^	Severely obese (≥35), *N* = 133^a^	*p*-value^b^
Age, years	63 (56, 70)	63 (61, 70)	63 (56, 70)	64 (57, 70)	64 (58, 69)	61 (53, 68)	0.026
Sex							<0.001
Female	440 (39%)	16 (73%)	162 (48%)	128 (30%)	74 (37%)	60 (45%)	
Male	680 (61%)	6 (27%)	174 (52%)	300 (70%)	127 (63%)	73 (55%)	
Race							<0.001
White	741 (66%)	9 (41%)	198 (59%)	296 (69%)	144 (72%)	94 (71%)	
Black	161 (14%)	8 (36%)	40 (12%)	51 (12%)	38 (19%)	24 (18%)	
Asian	18 (1.6%)	2 (9.1%)	8 (2.4%)	6 (1.4%)	2 (1.0%)	0 (0%)	
Other	8 (0.7%)	0 (0%)	4 (1.2%)	1 (0.2%)	2 (1.0%)	1 (0.8%)	
Unknown	192 (17%)	3 (14%)	86 (26%)	74 (17%)	15 (7.5%)	14 (11%)	
Ethnicity							0.3
Hispanic/Latino	76 (6.8%)	0 (0%)	22 (6.5%)	30 (7.0%)	17 (8.5%)	7 (5.3%)	
Not Hispanic/Latino	860 (77%)	19 (86%)	228 (68%)	328 (77%)	172 (86%)	113 (85%)	
Other	34 (3.0%)	2 (9.1%)	7 (2.1%)	16 (3.7%)	3 (1.5%)	6 (4.5%)	
Unknown	150 (13%)	1 (4.5%)	79 (24%)	54 (13%)	9 (4.5%)	7 (5.3%)	
Prior ASCT before first progression^c^	484 (43%)	12 (55%)	133 (40%)	187 (44%)	95 (47%)	57 (43%)	0.4
ASCT eligible	511 (46%)	10 (45%)	191 (57%)	196 (46%)	71 (35%)	43 (32%)	<0.001
Cytogenetic risk^d^							0.7
High risk	198 (18%)	3 (14%)	65 (19%)	75 (18%)	30 (15%)	25 (19%)	
Standard risk	554 (49%)	15 (68%)	158 (47%)	215 (50%)	104 (52%)	62 (47%)	
Unknown	368 (33%)	4 (18%)	113 (34%)	138 (32%)	67 (33%)	46 (35%)	
Immunoglobulin G							0.038
Yes	573 (51%)	14 (64%)	164 (49%)	203 (47%)	121 (60%)	71 (53%)	
No	276 (25%)	5 (23%)	76 (23%)	116 (27%)	43 (21%)	36 (27%)	
Unknown	271 (24%)	3 (14%)	96 (29%)	109 (25%)	37 (18%)	26 (20%)	
ECOG performance status							<0.001
0	294 (26%)	3 (14%)	81 (24%)	114 (27%)	61 (30%)	35 (26%)	
≥1	549 (49%)	15 (68%)	144 (43%)	211 (49%)	102 (51%)	77 (58%)	
Unknown	277 (25%)	4 (18%)	111 (33%)	103 (24%)	38 (19%)	21 (16%)	
ISS Stage							0.8
ISS1-2	782 (70%)	16 (73%)	230 (68%)	302 (71%)	148 (74%)	86 (65%)	
ISS3	309 (28%)	6 (27%)	96 (29%)	114 (27%)	50 (25%)	43 (32%)	
Unknown	29 (2.6%)	0 (0%)	10 (3.0%)	12 (2.8%)	3 (1.5%)	4 (3.0%)	
Treatment^e^							<0.001
Doublet	158 (14%)	6 (27%)	46 (14%)	61 (14%)	26 (13%)	19 (14%)	
Four or more	160 (14%)	4 (18%)	47 (14%)	62 (14%)	30 (15%)	17 (13%)	
Triplet (Bortezomib or Other)	597 (53%)	11 (50%)	153 (46%)	230 (54%)	123 (61%)	80 (60%)	
Triplet (Carfilzomib)	205 (18%)	1 (4.5%)	90 (27%)	75 (18%)	22 (11%)	17 (13%)	
CCI scores							0.21
≤2	92 (8.2%)	1 (4.5100%)	37 (11%)	26 (6·1%)	14 (7·0%)	14 (11%)	
>2 & <5	535 (48%)	9 (410%)	166 (49%)	202 (47%)	94 (47%)	64 (48%)	
≥5	493 (44%)	12 (55%0%)	133 (40%)	200 (47%)	93 (46%)	55 (41%)	
Frailty score							0.4
Frail	572 (68%)	155 (69%)	103 (63%)	79 (71%)	220 (68%)	15 (83%)	
Nonfrail	271 (32%)	70 (31%)	60 (37%)	33 (29%)	105 (32%)	3 (17%)	
Unknown	277	111	38	21	103	4	

^a^*n* (%); Median (IQR)

^b^Kruskal–Wallis rank sum test; Pearson’s Chi-squared test or Fisher’s exact test

^c^ASCT (specifically, any ASCT before the first progression event).

^d^Cytogenetic data were sub-classified as high-risk, standard risk, or unknown. Patients with a 17p deletion or t(4;14) or t(14;16) translocations were classified as high-risk; patients negative for all three abnormalities were classified as standard risk. If unknown or missing for any of the cytogenetic categories with no other high-risk abnormalities recorded, the patients’ cytogenetic risk was classified as unknown.

^e^The induction treatment combination variable had the categories of doublet (proteasome inhibitor or immunomodulatory drug [IMiD] with steroids), bortezomib based (bortezomib with steroids and a third drug, usually the IMiD lenalidomide), carfilzomib-based triplet (carfilzomib with steroids and a third drug, usually the IMiD lenalidomide), or other triplet, and quadruplet (four or more agents during induction)).

*BMI* Body Mass Index, *ASCT* Autologous Stem Cell Transplant, *ECOG* Eastern Cooperative Oncology Group, *ISS* International Staging System.

Underweight and severely obese patients had lower median PFS and OS than normal, overweight, and moderately obese patients. Multivariable models associating PFS and OS with BMI showed that underweight patients had a significantly higher risk of death (HR: 2.32; 95% CI: 1.09, 4.97). In addition, severely obese patients may have higher risk of progression (HR: 1.29; 95% CI: 0.99, 1.67) and death (HR: 1.43; 95% CI: 0.98-2.08) when compared to patients with normal BMI, although differences between groups did not achieve statistical significance (Table 2).

**Table 2:** Hazard ratios and 95% confidence intervals for the association of BMI with survival in newly diagnosed MM patients in the multiple myeloma research foundation CoMMpass registry.

	Number at risk (number of events)	Median^a^ time to event (months, 95% CI)	Multivariable adjusted^b^ HR (95% CI)
Progression-free survival			
Underweight (<18.5)	22 (14)	22.40 (16.39, NR)	1.45 (0.82, 2.55)
Normal (18.5 to <25)	336 (159)	34.56 (26.87, 43.66)	1 (ref)
Overweight (25 to <30)	428 (237)	32.13 (28.75, 36.27)	1.06 (0.87, 1.30)
Moderately obese (30 to <35)	201 (105)	36.63 (29.40, 48.98)	0.93 (0.73, 1.20)
Severely obese (>=35)	133 (93)	24.87 (20.53, 32.95)	1.29 (0.99, 1.67)
Overall survival			
Underweight (<18·5)	22 (8)	NR (36.20, NR)	2.32 (1.09, 4.97)
Normal (18.5 to <25)	336 (73)	NR (NR, NR)	1 (ref)
Overweight (25 to <30)	428 (107)	NR (72.50, NR)	1.03 (0.76, 1.40)
Moderately obese (30 to <35)	201 (57)	NR (58.74, NR)	1.21 (0.85, 1.72)
Severely obese (>=35)	133 (49)	NR (58.77, NR)	1.43 (0.98, 2.08)

^a^Estimated using the unadjusted Kaplan–Meier survival analysis.

^b^Estimated using Cox regression adjusted for age, race, sex, cytogenetic risk, ECOG, ISS, treatment, and ASCT (time-dependent).

*MM* Multiple Myeloma, *BMI* Body Mass Index, *HR* Hazard Ratio, *CI* Confidence Interval, *NR* Not Reached.

OS differed significantly between CCI groups (log rank *p* < 0.001; 3-year OS [95% CI], CCI ≥ 5: 65% [61%, 70%]; CCI > 2 & < 5: 87% [84%, 90%], CCI ≤ 2: 87% [79%, 96%]). OS was also significantly worse in the frail group compared to the nonfrail group (logrank *p* < 0.001; 3-year OS [95% CI], frail: 70% [66%, 74%], nonfrail: 88% [84%, 92%]). In the sensitivity analysis that included CCI as a covariate, there were comparable effects of BMI, with borderline significance for decreased OS in the underweight group compared to the normal group (HR 2.12, 95% CI: 0.99, 4.53). Strength and direction of associations were comparable in sensitivity analysis removing ECOG and age from the model and including frailty with decreased OS in underweight (HR 2.38, 95% CI: 1.11, 5.08).

In this comprehensive analysis of a prospective cohort with a median follow up of over 2 years, we found that being underweight was associated with a 132% higher risk of death from any cause. Given that age, ISS, CCI, and cytogenetic risk were not altered in underweight (vs. normal weight) patients, we speculate that the observed adverse outcomes in this subgroup maybe due to high ECOG (i.e., poor performance status) and disease-related weight loss. Additionally, being severely obese demonstrated a suggestive association with worse PFS and OS in newly diagnosed MM patients. This is biologically plausible and could reflect a worse ECOG performance status in the severely obese patients, which may render them less likely to tolerate full dose induction or transplant regimens. For example, we observed that participants in the newly diagnosed MM cohort were less likely to receive carfilzomib-based therapy if they had an elevated BMI. Another mechanism may be that of increased adipocytes in the bone marrow niche of severely obese patients. Higher BMI correlates with higher levels of bone marrow adipocytes, which in turn can provide a favorable microenvironment for MM cell growth [12], contributing to oncogenesis and MM disease progression [13]. MM cells co-cultured with adipocytes exhibit increased growth and adhesion [14].

The study in US veterans showed that overweight and obese patients had lower MM mortality compared to healthy weight patients, whereas underweight patients had higher MM mortality. This association between higher BMI and survival became non-significant after adjustment for weight loss in the year prior to diagnosis. Like the US Veteran cohort, we observed inferior survival among the small number of underweight patients. However, our study, which included a separate category for BMI ≥ 35 kg/m^2^, showed that severely obese patients also had a suggestion of worse prognosis compared to normal weight patients. It is notable that the MM patients in the CoMMpass registry were 61% male, whereas the veterans’ cohort was 98% male. Therefore, sex differences in the association of obesity with mortality may have contributed to the observed differences [15].

A unique strength of this study was the use of a large well-characterized cohort with information on clinical disease characteristics and treatment regimens. Study limitations include the lack of weight measurement prior to and during the study [10] and the use of BMI instead of newer body composition assessments [16]. However, BMI is the most widely available measure in epidemiologic studies and its use allows for comparisons across studies [8, 9]. We did not have data to evaluate potential confounding by weight loss prior to MM diagnosis, which has been shown to contribute to worse OS [10]. Additional limitations include the inability to evaluate differences in drug doses and treatment-emergent adverse events in relation to BMI and systematic missingness for some variables such as ECOG, which is a known issue with large multi-institutional databases.

This comprehensive examination of BMI and survival in newly diagnosed MM patients suggests that underweight and severe obesity are associated with worse survival. Future studies of weight trajectories and body composition may help clarify these observations. Additionally, clinical research to understand if patients with extreme BMI may benefit from weight management strategies to improve outcomes may be of importance.

## Supplementary information

BMI and MM Survival Supplemental Material

## Data Availability

The dataset analyzed during the current study is available in the MMRF CoMMpass Study repository [https://themmrf.org/finding-a-cure/our-work/the-mmrf-commpass-study/].
