# Supplementary material for: Extreme body mass index and survival in newly diagnosed multiple myeloma patients
Source: Blood Cancer J. 2023 Jan 12;13(1):13. doi: 10.1038/s41408-022-00782-7 (PMC9834289; doi:10.1038/s41408-022-00782-7)

**Supplemental Information** **– Extreme Body Mass Index and Survival in Newly Diagnosed Multiple Myeloma Patients**

eTable 1: Multivariable Cox Regression Model for PFS and OS in Patients with Newly Diagnosed MM

eFigure 1: Kaplan Meier Survival Curves in Newly Diagnosed MM

A] Progression Free Survival and B] Overall Survival

The missing category is not represented because there were only 15 patients.

eFigure 2: Overall Survival by Charlson Comorbidity Index (CCI) Score in Patients with Newly Diagnosed MM

eFigure 3: Overall Survival by Frailty Score in Patients with Newly Diagnosed MM

**eTable 1: Multivariable Cox Regression Model for PFS and OS** **in Patients with Newly Diagnosed MM**

| **PFS** | | | **OS** | | |  |  |
| --- | --- | --- | --- | --- | --- | --- | --- |
|  | **HR** | **95% CI** | **p-value** | **HR** | **95% CI** | **p-value** | |
| **BMI Categorized** |  |  |  |  |  |  |  |
| Normal | — | — |  | — | — |  |  |
| Underweight | 1.45 | 0.82, 2.55 | 0.2 | 2.32 | 1.09, 4.97 | 0.030 |  |
| Overweight | 1.06 | 0.87, 1.30 | 0.6 | 1.03 | 0.76, 1.40 | 0.8 |  |
| Moderately Obese (≥ 30 & < 35) | 0.93 | 0.73, 1.20 | 0.6 | 1.21 | 0.85, 1.72 | 0.3 |  |
| Severely Obese (≥ 35) | 1.29 | 0.99, 1.67 | 0.058 | 1.43 | 0.98, 2.08 | 0.062 |  |
| **Age (per 1 year increase)** | 1.01 | 1.00, 1.02 | 0.018 | 1.02 | 1.01, 1.04 | <0.001 |  |
| **Race** |  |  |  |  |  |  |  |
| White | — | — |  | — | — |  |  |
| Black | 1.08 | 0.87, 1.34 | 0.5 | 1.22 | 0.91, 1.64 | 0.2 |  |
| Asian | 1.25 | 0.69, 2.25 | 0.5 | 0.55 | 0.17, 1.75 | 0.3 |  |
| Other | 1.64 | 0.61, 4.44 | 0.3 | 1.24 | 0.30, 5.05 | 0.8 |  |
| Unknown | 0.69 | 0.47, 1.01 | 0.059 | 0.63 | 0.33, 1.20 | 0.2 |  |
| **Cytogenetic Risk** |  |  |  |  |  |  |  |
| High Risk | — | — |  | — | — |  |  |
| Low Risk | 0.74 | 0.59, 0.93 | 0.010 | 0.68 | 0.49, 0.93 | 0.015 |  |
| Unknown | 0.96 | 0.76, 1.21 | 0.7 | 0.73 | 0.52, 1.02 | 0.069 |  |
| **ECOG Performance Status** |  |  |  |  |  |  |  |
| 0 | — | — |  | — | — |  |  |
| ≥1 | 1.43 | 1.17, 1.74 | <0.001 | 1.61 | 1.19, 2.17 | 0.002 |  |
| Unknown | 1.41 | 1.07, 1.86 | 0.015 | 1.36 | 0.89, 2.08 | 0.2 |  |
| **Sex** |  |  |  |  |  |  |  |
| Female | — | — |  | — | — |  |  |
| Male | 1.16 | 0.98, 1.37 | 0.094 | 1.64 | 1.27, 2.12 | <0.001 |  |
| **ISS** |  |  |  |  |  |  |  |
| ISS1-2 | — | — |  | — | — |  |  |
| ISS3 | 1.39 | 1.16, 1.66 | <0.001 | 2.05 | 1.61, 2.62 | <0.001 |  |
| Unknown | 1.55 | 0.98, 2.45 | 0.063 | 1.24 | 0.60, 2.54 | 0.6 |  |
| **Treatment** |  |  |  |  |  |  |  |
| Doublet | — | — |  | — | — |  |  |
| Four Or More | 0.67 | 0.50, 0.89 | 0.005 | 0.47 | 0.31, 0.73 | <0.001 |  |
| Triplet (Bortezomib or Other) | 0.82 | 0.66, 1.02 | 0.078 | 0.76 | 0.56, 1.02 | 0.067 |  |
| Triplet (Carfilzomib) | 0.60 | 0.40, 0.88 | 0.009 | 0.42 | 0.22, 0.80 | 0.008 |  |
| **ASCT (time dependent)** | 0.47 | 0.39, 0.58 | <0.001 | 0.57 | 0.42, 0.76 | <0.001 |  |
| Abbreviations: PFS = Progression Free Survival, OS = Overall Survival, BMI = Body Mass Index, HR = Hazard Ratio, CI = Confidence Interval ECOG = Eastern Cooperative Oncology Group, ISS = International Staging System, ASCT = Autologous Stem Cell Transplant. | | | | | | |  |

**eFigure 1: Kaplan Meier Survival Curves in Newly Diagnosed MM**

**A] Progression Free Survival and B] Overall Survival**

**The missing category is not represented because there were only 15 patients.**

**A]**


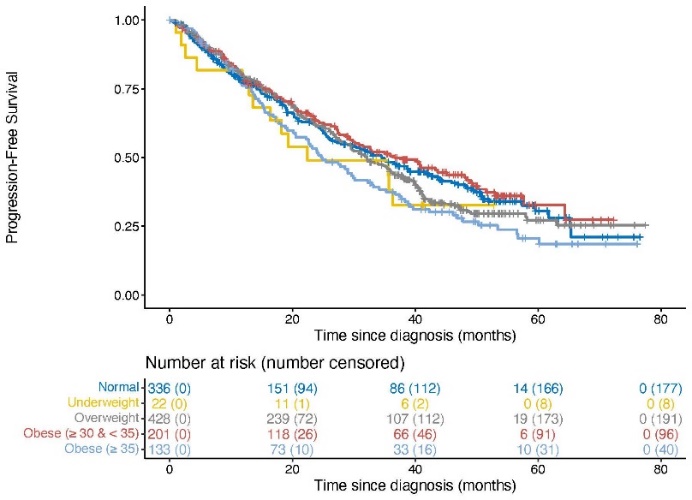


**B]**


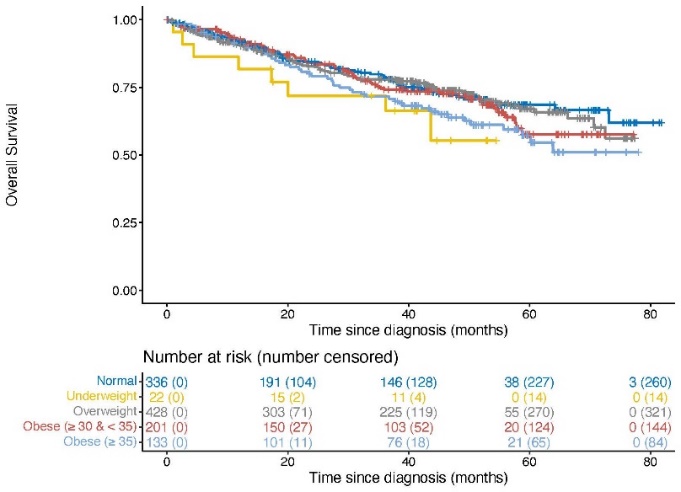


**eFigure 2: Overall Survival by** **Charlson Comorbidity Index (CCI)** **Score in Patients with Newly Diagnosed Multiple Myeloma**


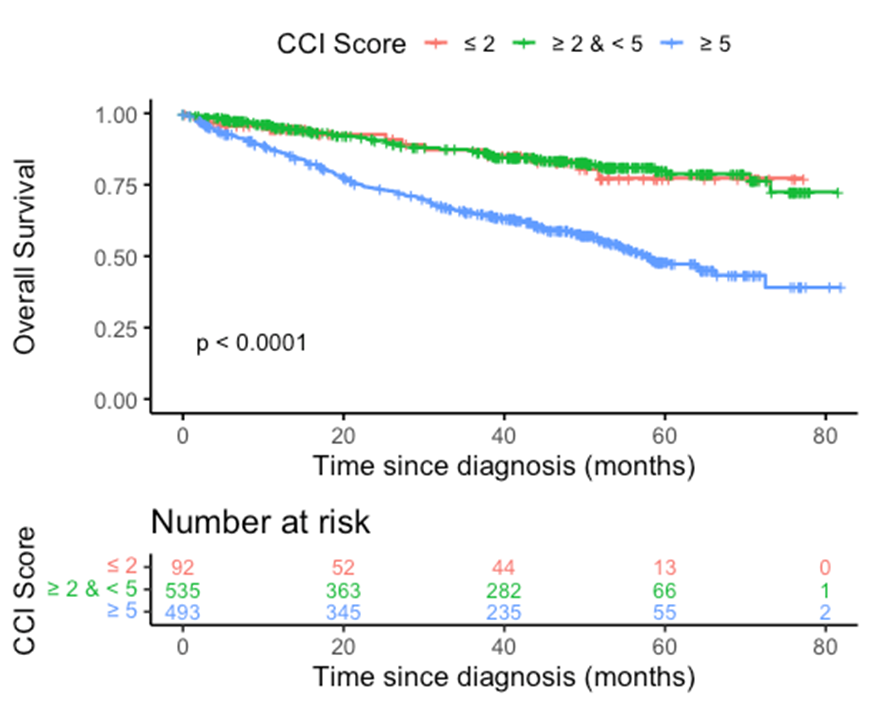


Log Rank p <0.0001

**eFigure 3: Overall Survival by Frailty Score in Patients with Newly Diagnosed Multiple Myeloma**


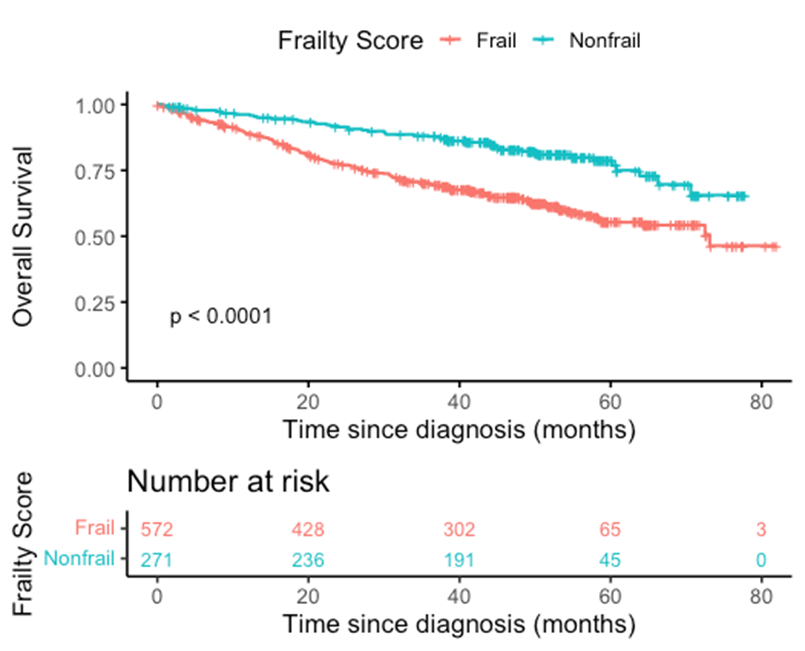

Supplement: Supplementary file 1 — BMI and MM Survival Supplemental Material [file 41408_2022_782_MOESM1_ESM.docx]
